# High Incidence of Active Tuberculosis in Asylum Seekers from Eritrea and Somalia in the First 5 Years after Arrival in the Netherlands

**DOI:** 10.3201/eid2604.190123

**Published:** 2020-04

**Authors:** Jossy van den Boogaard, Erika Slump, Henrieke J. Schimmel, Wim van der Hoek, Susan van den Hof, Gerard de Vries

**Affiliations:** European Programme for Intervention Epidemiology Training, European Centre for Disease Prevention and Control, Stockholm, Sweden (J. van den Boogaard);; National Institute for Public Health and the Environment, Bilthoven, the Netherlands (J. van den Boogaard, E. Slump, H.J. Schimmel, W. van der Hoek, S. van den Hof, G. de Vries)

**Keywords:** tuberculosis and other mycobacteria, TB, incidence, asylum seekers, Eritrea, Somalia, Netherlands, screening, latent tuberculosis infection, immigration, bacterial infections

## Abstract

Three quarters of tuberculosis (TB) patients in the Netherlands are foreign-born; 26% are from Eritrea or Somalia. We analyzed TB incidence rates in asylum seekers from Eritrea and Somalia in the first 5 years after arrival in the Netherlands (2013–2017) and performed survival analysis with Cox proportional hazards regression to analyze the effect of age and sex on the risk for TB. TB incidence remained high 5 years after arrival in asylum seekers from Eritrea (309 cases/100,000 person-years) and Somalia (81 cases/100,000 person-years). Age >18 years was associated with a higher risk for TB in asylum seekers from Eritrea (3.4 times higher) and Somalia (3.7 times higher), and male sex was associated with a 1.6 times higher risk for TB in asylum seekers from Eritrea. Screening and treating asylum seekers from high-incidence areas for latent TB infection upon arrival would further reduce TB incidence in the Netherlands.

The Netherlands is a low-incidence country for tuberculosis (TB). The TB notification rate was 4.6 cases/100,000 population in 2017, the lowest ever recorded in the country. Most TB patients in the Netherlands are immigrants and asylum seekers. In 2017, of the Netherlands’ 787 TB patients, 74% were foreign-born; persons from Eritrea and Somalia together accounted for 26% of all foreign-born patients (*1*). In line with the World Health Organization (WHO) End TB Strategy and the related framework and plans toward TB elimination in low-incidence countries, the Netherlands is aiming to reduce TB incidence by 25% in the next 5 years (*2*–*5*). The ultimate aim is to reach the preelimination phase for TB (<1 TB patient/100,000 population/year) and subsequently elimination (<1 TB patient/1 million population/year). One of the priority actions for low-incidence countries to proceed toward this goal is to have screening programs in place for active TB and latent TB infection (LTBI) in selected high-risk groups, such as asylum seekers from high-incidence countries (*4*).

Nearly one fourth of the world population has LTBI, which is especially prevalent among those living in countries with high incidence of active TB (*6*). LTBI refers to a persistent host immune response to *Mycobacterium tuberculosis* antigens without evidence of clinically manifest active TB. Persons with LTBI generally have no symptoms of TB but are at risk for active TB. This risk is highest in the first 2 years after infection. Unlike active TB, which can usually be diagnosed on the basis of a combination of signs and symptoms, imaging (e.g., chest radiograph), and bacteriologic examination, LTBI is diagnosed by tuberculin skin test and interferon-γ release assays. Therefore, screening programs for active TB differ from those for LTBI. Treatment of LTBI, which typically requires fewer antibiotic drugs over a shorter period compared with active TB, can prevent future onset of active TB and transmission of the disease (*7*).

In the Netherlands, asylum seekers and immigrants from countries with a WHO-estimated TB incidence of >50 cases/100,000 population and who have an intended stay in the Netherlands of >3 months undergo mandatory screening for active TB. Asylum seekers are screened within the first 3 days after reporting to a reception center; immigrants are screened usually within the first 3 months of arrival in the country. Furthermore, asylum seekers and immigrants from countries with a WHO-estimated TB incidence of >200 cases/100,000 population (e.g., Somalia, which has an incidence of 270 cases/100,000 population) (*8*) or from countries with a high TB prevalence at entry screening in the Netherlands (e.g., Eritrea, which has a WHO-estimated incidence of 74 cases/100,000 population but a prevalence of >280 cases/100,000 population at entry screening in the Netherlands) (*8*,*9*) are offered biannual follow-up screening by chest radiograph during the first 2 years after arrival (*5*,*9*). The coverage of entry screening of asylum seekers is nearly 100%. However, the coverage of follow-up screening drops to 14% at 6 months after entry and 6% at 2 years after entry. A recent 5-year evaluation of the Netherlands’ TB screening program for asylum seekers concluded that radiologic follow-up screening is not effective, because of its low coverage. Only 36% of TB patients who were eligible for follow-up screening and did not have TB diagnosed at entry screening were found through follow-up screening (*10*).

Replacing radiologic follow-up screening with an LTBI screening and treatment program for asylum seekers from high-incidence countries upon arrival in the Netherlands is likely to be more effective in reaching the targets set by the Netherlands’ TB Control Strategy. Such screening would identify persons at risk for active TB in the future and provide opportunities to prevent the disease. The Netherlands’ Committee for Practical TB Control already recommended replacing radiologic screening with LTBI screening for immigrants and asylum seekers <18 years of age. As of the publication date of this article, this approach was being implemented for immigrants <18 years of age but not yet for asylum seekers (*5*,*9*). To inform policy makers and professionals on the potential benefit of an LTBI screening program for asylum seekers from high-incidence countries, we analyzed trends in TB incidence rates in asylum seekers from Eritrea and Somalia in the first 5 years after their arrival in the Netherlands.

## Methods

We performed a retrospective cohort study in asylum seekers from Eritrea and Somalia who arrived in the Netherlands from January 1, 2013, through December 31, 2017. Because Eritrea was not yet an independent country before 1991, persons from Eritrea who were born before 1991 were actually born in Ethiopia. Because our data sources reported on country of birth (instead of nationality), we combined the groups of asylum seekers from Eritrea whose country of birth was listed as Eritrea or Ethiopia (3.4% persons in this combined group were born in Ethiopia).

We obtained data from the Netherlands’ Immigration and Naturalization Service (IND) on asylum seekers from Eritrea and Somalia arriving in the Netherlands during the study period. IND provided data on numbers of asylum seekers and reason of request (e.g., first applications, repeated applications, family reunifications, and invited asylum seekers) by country of birth, month and year of arrival, sex, and age group. We excluded repeated applications because these are usually from asylum seekers who reapply after a failed first application without leaving the country in between applications. The IND does not provide information on duration of stay in the Netherlands of asylum seekers after registration (i.e., no linked data of asylum seekers who left the country or died are available). Because asylum seekers from Eritrea and Somalia are usually granted asylum in the Netherlands and therefore stay in the country (*11*), we assumed that the follow-up period of each person in the study population lasted either until the end of the study period (December 31, 2017) or until the event (TB diagnosis) occurred.

We obtained data from the Netherlands’ National TB Register (NTR) on TB patients from Eritrea and Somalia who arrived in the Netherlands during the study period (i.e., persons diagnosed with active TB through December 31, 2017). NTR contains detailed information on patient demographics (including date of arrival in the Netherlands, age, sex, and country of birth), diagnostic and disease characteristics, and treatment outcome. NTR also has information on whether patients belong to specific risk groups, such as asylum seekers or immigrants. Because asylum seekers who have onset of TB after obtaining asylum are registered as immigrants in NTR, we included asylum seekers and immigrants from NTR as long as they had arrived on or after January 1, 2013. We excluded TB patients with unknown date of arrival in the Netherlands (n = 41).

We defined cases of prevalent TB as active TB in patients who were registered in NTR as being found through entry screening, independent of the time that had passed since arrival. We defined cases of incident TB as active TB in patients who were not found through entry screening, including patients who self-reported symptoms and patients found through follow-up screening or contact tracing. Patients with pulmonary and extrapulmonary TB were included. Because entry screening is conducted by using chest radiograph, mainly pulmonary and some forms of intrathoracic TB (e.g., pleural TB) are detected by screening. TB diagnosis follows the criteria set by European Union member states (i.e., cases are confirmed if *M. tuberculosis* is found in patient specimens; cases are deemed probable or possible if bacteriologic or clinical criteria are met) (*12*).

We merged both datasets (the cohort of asylum seekers from Eritrea and Somalia who arrived during the study period and the TB patients registered in NTR) by month and year of arrival, country of birth, sex, and age. We calculated the total follow-up time (in person-months) for cases by subtracting the date of arrival from the date of diagnosis, and for noncases by subtracting the estimated date of arrival (set at the 15th day of the registered month of arrival) from December 31, 2017.

We analyzed data by using the statistics software package Stata/SE 15.1 (https://www.stata.com). We described and compared characteristics of (prevalent and incident) cases and noncases and calculated incidence rates by number of years after arrival. Because we were interested in the risk for TB over time in persons with different follow-up periods, we performed survival analysis with Cox proportional hazards regression. We calculated cumulative incidences and analyzed the effect of country of birth, calendar year of entry, age, and sex on the risk for active TB.

## Results

### Characteristics of the Study Population

The study population consisted of 26,057 persons (21,182 [81%] asylum seekers from Eritrea and 4,875 [19%] from Somalia (Table 1). The number of asylum seekers from Eritrea and Somalia arriving per calendar year varied. Whereas the peak of asylum seekers from Eritrea arriving in the Netherlands occurred in 2015, the arrival of asylum seekers from Somalia peaked in 2013 (and the years before), resulting in different median follow-up periods of the study population: 27 months (interquartile range 13–32 months) for those from Eritrea and 49 months (interquartile range 39–53 months) for those from Somalia. Asylum seekers from Eritrea were more often >18 years of age (65%) than those from Somalia (30%) (p<0.001). The proportion of men and boys in the study population from Eritrea was higher than that in the study population from Somalia (61% vs. 48%; p < 0.001).

**Table 1:** Characteristics of asylum seekers from Eritrea and Somalia in whom active TB was detected, the Netherlands, 2013–2017

Characteristic	**Total study population**	**Country of origin**		**p value†**
		Eritrea	Somalia	
Population size, no.	26,057	21,182	4,875	NA
Arrivals per year				
2013	3,741 (14.4)	911 (4.3)	2,830 (58.1)	<0.001
2014	5,353 (20.5)	4,168 (19.7)	1,185 (24.3)	<0.001
2015	8,889 (34.1)	8,378 (39.6)	511 (10.5)	<0.001
2016	3,484 (13.4)	3,250 (15.3)	234 (4.8)	<0.001
2017	4.590 (17.6)	4,475 (21.1)	115 (2.4)	<0.001
Age group				
<18 y	10,750 (41.3)	7,320 (34.6)	3,430 (70.4)	<0.001
>18 y	15,307 (58.7)	13,862 (65.4)	1,445 (29.6)	<0.001
Sex				
F	10,731 (41.2)	8,191 (38.7)	2,520 (51.7)	<0.001
M	15,326 (58.8)	12,991 (61.3)	2,355 (48.3)	<0.001
Persons with prevalent TB	78 (0.3)	61 (0.3)	17 (0.4)	0.48
Of whom had PTB	59/78 (75.6)	49/61 (80.3)	10/17 (62.5)	0.068
Persons with incident TB	468 (1.8)	338 (1.6)	130 (2.7)	<0.001‡
Of whom had PTB	238/468 (50.9)	181/338 (53.6)	57/130 (43.5)	0.060
Detected in follow-up screening	77/468 (16.5)	67/338 (19.8)	10/130 (7.6)	0.002

*Values are no. (%) unless indicated. NA, not applicable; PTB, pulmonary tuberculosis; TB, tuberculosis. †Case-patients from Eritrea compared with those from Somalia. ‡Proportion of incident cases cannot truly be compared between case-patients from Eritrea and those from Somalia because follow-up time was different (median of 28 months for case-patients from Eritrea versus 49 months for those from Somalia).

A total of 546 TB patients were identified. Seventy-eight patients (61 from Eritrea and 17 from Somalia) had prevalent TB found through entry screening, indicating a TB prevalence at entry of 288 (95% CI 224–370) cases/100,000 population for asylum seekers from Eritrea and 349 (95% CI 217–560) cases/100,000 population for those from Somalia. The other 468 patients had incident TB (338 were from Eritrea and 130 from Somalia), corresponding to overall incidence rates of 747 (95% 672–831) cases/100,000 population for asylum seekers from Eritrea and 712 (95% CI 600–846) cases/100,000 population for those from Somalia. Sixteen percent of incident cases were identified through follow-up screening. The proportion of pulmonary TB was higher among patients identified through entry screening than those identified after arrival (76% vs. 51%; p<0.001).

### Incidence Rates over Time and Survival Analysis

**Figure 1:**
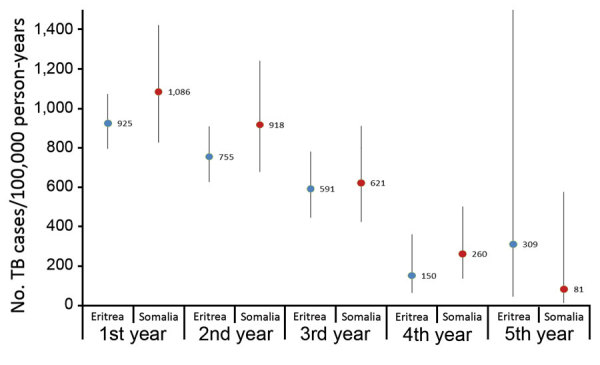
Trend of TB incidence rates (cases/100,000 person-years) of asylum seekers arriving from Eritrea and Somalia in the Netherlands, 2013–2017, by year after arrival. Error bars indicate 95% CIs; upper limit of the 95% CI for persons from Eritrea in the fifth year after arrival (2017) is 2,195. TB, tuberculosis.

We determined the trend in TB incidence rate over the first 5 years after arrival in the Netherlands, stratified by country of birth (Figure 1). Among asylum seekers from Eritrea, the incidence rate dropped from 925 (95% CI 796–1,073) cases/100,000 person-years in the first year after arrival to 150 (95% CI 62–360) cases/100,000 person-years in the fourth year after arrival and to 309 (95% CI 44–2,195) cases/100,000 person-years in the fifth year after arrival. For asylum seekers from Somalia, the incidence rate dropped from 1,086 (95% CI 828–1,425) cases/100,000 person-years in the first year after arrival to 260 (95% 135–500) cases/100,000 person-years in the fourth year after arrival and to 81 (95% CI 11–575) cases/100,000 person-years in the fifth year after arrival. The large 95% CI around the fifth year incidence rate in asylum seekers from Eritrea reflects the small number of this population that had been followed up in this study for 5 years.

**Figure 2:**
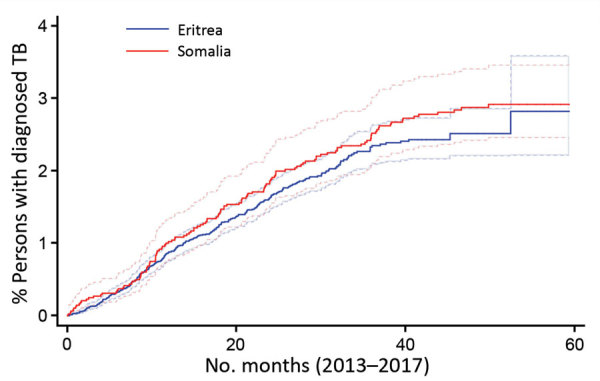
Kaplan-Meier curve indicating risk for TB among asylum seekers arriving from Eritrea and Somalia in the Netherlands, over a 60-month follow-up period (2013–2017). TB, tuberculosis.

Figure 2 depicts the Kaplan-Meier curve for onset of TB over time. The cumulative risk for TB was ≈3% over the first 5 years after arrival in both groups.

### Effect of Age and Sex

When stratified by country of birth, age >18 years was associated with a higher risk for TB in the study population (hazard ratio 3.4 [95% CI 2.4–4.9] in asylum seekers from Eritrea and 3.7 [95% CI 2.6–5.3] in those from Somalia) (Table 2). In asylum seekers from Eritrea, male sex was also associated with a higher risk for TB (hazard ratio 1.6 [1.3–2.1]); in those from Somalia, no association with sex was found. Because of strong (graphic) evidence against the proportional hazards assumption for the variable calendar year of arrival, we further stratified by this variable (data not shown). The effect of age and sex in a model with stratification by country of birth and calendar year of arrival appeared similar to the model with stratification by country of birth only but was not statistically significant because of small numbers in each stratum.

**Table 2:** Results of Cox proportional hazards regression analysis indicating hazard ratios for age and sex, stratified by country of birth, among asylum seekers from Eritrea and Somalia with incident tuberculosis cases, the Netherlands, 2013–2017

Country of origin and characteristic	**Total**	**No. cases (%)**		**Hazard ratio (95% CI)**	
Eritrea					
Age group					
<18 y	7,301	35 (0.5)		Referent	
>18 y	13,820	303 (2.2)		3.4 (2.4–4.9)	
Sex					
F	8,185	86 (1.1)		Referent	
M	12,936	252 (2.0)		1.6 (1.3–2.1)	
Somalia					
Age group					
<18 y	3,419	54 (1.6)		Referent	
>18 y	1,439	76 (5.3)		3.7 (2.6–5.3)	
Sex					
F	2,513	74 (2.9)		Referent	
M	2,345	56 (2.4)		1.0 (0.7–1.4)	

## Discussion

Our study showed that asylum seekers in the Netherlands from Eritrea and Somalia have a high risk for TB: 0.3% had TB upon arrival, and ≈3% had onset of TB in the first 5 years after arrival. Although incidence rates gradually declined, they were still 10–50 times higher than the overall TB incidence in the Netherlands. Furthermore, our study provides additional insight in specific risk groups for active TB: adult (mainly those 18–35 years old) asylum seekers from Eritrea and Somalia were at higher risk for TB compared with those <18 years of age, as were men and boys from Eritrea compared with women and girls from Eritrea.

These results are consistent with a study conducted in the Netherlands in 2004, which showed that the incidence of pulmonary TB remained high in immigrants from high-incidence countries at least a decade after arrival in the Netherlands (*13*). A study in Denmark showed that in the 1990s, the annual incidence of TB in immigrants from Somalia decreased only gradually during the first 7 years of residence, from 2,000 to 700 cases/100,000 population (*14*). Although these studies provide data on changes in incidence over time, we used survival analysis methods to analyze and depict the risks of a cohort of newly arrived asylum seekers, which enabled us to take into account the different follow-up periods for each person in the study.

The TB prevalence and incidence rates in asylum seekers from Eritrea and Somalia in our study were much higher than the WHO-estimated TB incidence in Eritrea (74 cases/100,000 population) and in Somalia (270 cases/100,000 population) (*8*). A plausible explanation for this finding is the additional risk for infection while traveling to Europe, where overcrowding and unsanitary conditions are common along travel routes, on top of the baseline infection risk in the country of birth (*15*,*16*). Walker et al. (*17*) found molecular and epidemiologic evidence for this in their study of a cluster of multidrug-resistant *M. tuberculosis* infections among patients arriving in Europe from the Horn of Africa. A second explanation could be an increased risk for TB because of vitamin D deficiency, malnutrition, and stress (*15*). These conditions are common in asylum seekers during the often stressful asylum application procedures and during the first years of settlement in the new country. Third, transmission within ethnic groups in the new country of residence can also contribute to the higher TB rates found in asylum seekers. Occasional outbreaks have been reported in ethnic groups, including recently arrived asylum seekers (*18*). Finally, the WHO figures could be an underestimation of actual TB rates (*18*–*20*).

Our findings support the recommendations for LTBI screening of asylum seekers from high TB-incidence countries (*4*). In our study, most TB cases in asylum seekers from Eritrea and Somalia occurred after initial radiologic screening for active TB, and only a few cases were identified by radiologic follow-up screening (partly because of the low coverage of follow-up screening). LTBI screening and treatment can prevent active TB, including extrapulmonary forms. Furthermore, because LTBI might reactivate to active disease many years after infection, LTBI screening also has the potential to prevent TB in asylum seekers many years and even decades after arrival in the new country. Implementing an LTBI screening program in asylum seekers is not easy. A survey conducted by the WHO Regional Office for Europe and the European Respiratory Society showed that 53% of countries in Europe performed systematic LTBI screening in refugee populations (*21*). A study conducted in 11 selected countries of Europe indicated that these countries had very different methods and policies for migrant TB or LTBI screening (*22*). Systematic reviews have demonstrated that limited information is available on the yield and effectiveness of migrant LTBI screening (*22**,**23*). Furthermore, cost-effectiveness of LTBI screening as predicted in mathematical models is highly setting-specific, with best results achieved if restricted to migrants from high-incidence countries (*24*).

Our study had several strengths. We were able to combine national comprehensive data on immigration and TB notification; thus, we could calculate incidence rates for specific groups of asylum seekers and show trends over time after arrival. Moreover, by using a Cox proportional hazards regression model, we were able to assess the effect of risk factors such as sex and age on the risk for TB. 

Our study also had some limitations. First, no data were available on the actual follow-up period of persons in the study population who did not have TB diagnosed. Although most asylum applicants from Eritrea and Somalia are granted asylum and will therefore stay in the Netherlands (*11*), some might have moved to another country (or died) before the end of our study period, meaning that we possibly underestimated the TB incidence rates in asylum seekers from Eritrea and Somalia. On the other hand, whereas we included only asylum seekers (not immigrants) from the IND database, we did not differentiate between asylum seekers and immigrants among the TB patients included in our study. The reason for this was that asylum seekers who obtained a residence permit before having their TB diagnosed are registered in NTR as immigrants, even though most arrived in the Netherlands as asylum seekers. Very few asylum seekers from Eritrea and Somalia come to the Netherlands as immigrants (*11*,*25*), so we expect that this limitation has not led to a substantial overestimation of TB incidences in our study. Second, no information on travel routes was available for our study population, and no distinction could be made between asylum seekers who had undertaken the often long and stressful journey by land and water and those who came directly by airplane, for example, for the purpose of family reunification. Future studies should take these differences into account. Moreover, we only analyzed data on asylum seekers from 2 countries with high TB incidence countries (Eritrea and Somalia), so the results might not reflect trends in onset of active TB in asylum seekers from other high-incidence countries. However, because asylum seekers from other countries in Africa often share the same hazardous journey, their risk for TB is probably similarly elevated. An investigation into whether the risks for active TB after arrival in the Netherlands differ between asylum seekers and other migrants from high-incidence countries (e.g., persons migrating to the Netherlands because of work or study) would be warranted. We recommend additional studies of longer follow-up periods to enable a more extensive analysis of trends in TB incidence rates, molecular studies differentiating disease caused by in-country transmission or reactivation of premigration acquired infection, and studies evaluating the effectiveness and impact of LTBI screening programs in asylum seekers.

In conclusion, our study results clearly show that asylum seekers from Eritrea and Somalia remain at high risk for active TB for at least the first 5 years after arrival in the Netherlands. This finding underscores the need for an LTBI screening and treatment program for high-risk groups. LTBI screening and preventive treatment will also accelerate TB control and contribute toward the elimination of TB.
